# Physical training in adults with asthma: An integrative approach on strategies, mechanisms, and benefits

**DOI:** 10.3389/fresc.2023.1115352

**Published:** 2023-02-17

**Authors:** Fabiano Francisco de Lima, David Halen Araújo Pinheiro, Celso Ricardo Fernandes de Carvalho

**Affiliations:** Department of Physical Therapy, School of Medicine, University of Sao Paulo, Sao paulo, Brazil

**Keywords:** asthma, exercise training, symptoms, inflammation, quality of life

## Abstract

Asthma is a chronic airway disease characterized by airflow limitation and respiratory symptoms associated with chronic airway and systemic inflammation, bronchial hyperreactivity (BHR), and exercise-induced bronchoconstriction (EIB). Asthma is a heterogeneous disease classified according to distinct airway and systemic inflammation. Patients commonly present with several comorbidities, including anxiety, depression, poor sleep quality, and reduced physical activity levels. Individuals with moderate to severe asthma often have more symptoms and difficulty achieving adequate clinical control, which is associated with poor quality of life, despite proper pharmacological treatment. Physical training has been proposed as an adjunctive therapy for asthma. Initially, it was suggested that the effect of physical training might be attributed to the improved oxidative capacity and reduced production of exercise metabolites. However, in the last decade, there has been evidence that aerobic physical training promotes anti-inflammatory effects in asthma patients. Physical training improves BHR and EIB, asthma symptoms, clinical control, anxiety, and depression levels, sleep quality, lung function, exercise capacity, and dyspnea perception. Furthermore, physical training reduces medication consumption. The most commonly used exercise strategies are moderate aerobic and breathing exercises; however, other techniques, such as high-intensity interval training, have shown promising effects. In the present study, we reviewed the strategies and beneficial effects of exercise on clinical and pathophysiological asthma outcomes.

## Introduction

Asthma is a chronic airway disease characterized by airflow limitation associated with respiratory symptoms such as wheezing, dyspnea, chest tightness, and coughing that vary over time with respect to its occurrence, frequency, and intensity. These variations are often triggered by factors such as exercise, exposure to allergens and/or irritants, weather changes, or viral respiratory infections. Asthma is usually associated with chronic airway inflammation and bronchial hyperresponsiveness (BHR), which may persist even when symptoms are absent or when lung function is normal and can normalize with treatment (1). As a result, the disease is highly prevalent and has morbidity, affecting up to 18% of the population in some countries (2).

Asthma treatment should be initiated immediately after diagnosis to control symptoms and reduce the risk of exacerbation. Treatment includes medication prescriptions (long-acting bronchodilators and inhaled corticosteroids) and non-pharmacological therapies. Among all non-pharmacological treatments, breathing exercises and physical training are considered to have the highest level of evidence (1, 3). Interestingly, despite the evidence on the effect of exercise, the Global Initiative for Asthma (GINA) still names it as physical activity. In addition, patients should participate in an educational program that covers information on disease pathophysiology, how to detect and avoid trigger factors, proficiency in inhaler use, adherence, written action plan for asthma, self-monitoring, and regular medical review (1).

Despite advances in pharmacological treatment, patients with moderate to severe asthma often present symptoms and difficulty achieving adequate clinical control. Therefore, GINA suggests non-pharmacological therapies, such as physical activity, breathing exercises, avoidance of indoor allergens, and smoking cessation. Among these non-pharmacological interventions, several systematic reviews have demonstrated the importance of physical training as an essential part of asthma treatment (4, 5). A recent study observed that both techniques (aerobic training or breathing exercise) induced similar effects on clinical control, quality of life (QoL), asthma symptoms, psychological distress, physical activity levels, and airway inflammation in patients with moderate to severe asthma (6). However, the number of studies demonstrating the mechanisms induced by exercise training is much larger. Historically, there was no consensus on the recommendation of physical training for patients with asthma until the beginning of 2000 because most patients have a unique response to exercise that can trigger exercise-induced bronchoconstriction (EIB) (7). EIB is a transitory condition that affects 40% to 90% of asthma patients and causes transitory airway narrowing during or after exercise (8). Evidence suggests that EIB intensity is related to disease severity (9), explaining why patients with moderate to intense asthma avoid exercise.

Despite the advances in recent years, the recommendation of physical exercise for individuals with asthma is still limited because of patients' fear of experiencing EIB. In 2000, Ram et al. (10) published the first systematic review on this subject, demonstrating the benefits of exercise training and reducing the paradigm for recommending physical training for patients with asthma. However, since 2000, several studies have suggested that physical training can reduce EIB, bronchial hyperresponsiveness, medication consumption, and airway and systemic inflammation and improve health factors related to QoL, aerobic potency, and clinical asthma control (5). In this integrative review, we collate all evidence demonstrating the effects of exercise on several previously mentioned asthma outcomes, including lung function, anxiety, and depression symptoms. Furthermore, we described the strategies of exercise training available for adults with asthma.

### Symptoms and clinical control

Asthma is associated with respiratory symptoms, such as wheezing, dyspnea, chest tightness, and coughing, which vary over time in frequency, occurrence, and intensity. In addition, symptoms are often triggered by factors such as exercise, exposure to allergens and/or irritants, weather changes, and viral respiratory infections (2). Careful asthma control in childhood is important, as asthma recurs in patients with morbidities and comorbidities in adulthood and determines lung function (11). However, when the onset of symptoms occurs during adulthood, it is estimated that 5%–20% of new cases are associated with occupational exposure, requiring more detailed investigation (12).

In patients older than 17 years, clinical asthma control can be assessed using the Asthma Control Questionnaire (ACQ), a simple and easy-to-use tool widely applied in several clinical trials worldwide (13). ACQ consists of five questions about asthma symptoms, one about rescue medication (*β*2-agonist), and the seventh question considers the predicted percentage value of forced expiratory volume in the first second (FEV_1_) (14). Volbeda et al. (2013) (15) observed that patients with well-controlled asthma had reduced BHR, exhaled nitric oxide levels, and eosinophilic inflammation; improved airway epithelial integrity; and better QoL scores than those with uncontrolled asthma. Systematic reviews have shown that aerobic training reduces disease symptoms and improves clinical control (5, 16). In addition, studies have shown that physical exercise effectively reduces the number of exacerbations (17–19). For example, França-Pinto et al. (2015) (17) reported that aerobic training reduced symptoms and the number of exacerbations in patients with asthma evaluated using the ACQ. In contrast, worsening symptoms and clinical control may favor an increase in exacerbations. Physical training programs have been used as a non-pharmacological intervention to reduce the severity of asthma episodes by helping to achieve adequate lung ventilation and improving QoL.

### Exercise capacity

**Figure 1:**
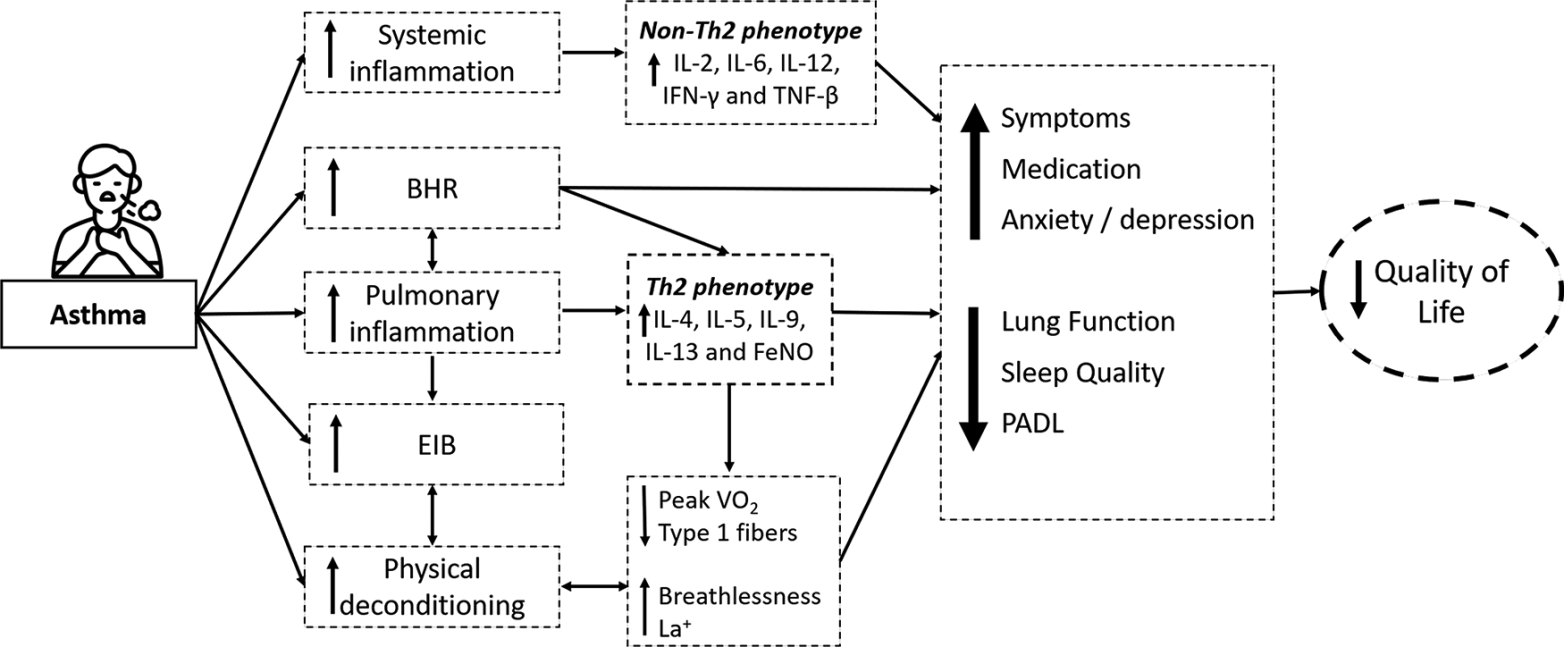
Illustrative figure presenting clinical outcomes, inflammatory mechanisms, and comorbidities in individuals with asthma. BHR: Bronchial hyperresponsiveness; EIB: exercise-induced bronchoconstriction; IL: interleukins; IFN-y: Interferon-gamma; TNF-*β*: Tumor Necrosis Factor, FeNO: Fractional Exhaled Nitric Oxide; PeakVO_2_: Peak oxygen consumption; La^+^: Blood lactate during moderate exercise; PADL: Physical Activity in Daily Life; Icon made by AomAm from www.flaticon.com.

Exercise capacity is impaired in patients with asthma (20). Shortness of breath can lead to physical inactivity, which can lead to physical deconditioning being a limiting factor for exercise capacity (21). A reduced physical fitness may limit daily performance more than airflow limitation (22) (Figure 1). Indeed, exercise training is well-tolerated by people with asthma and can improve cardiopulmonary fitness (23, 24).

**Figure 2:**
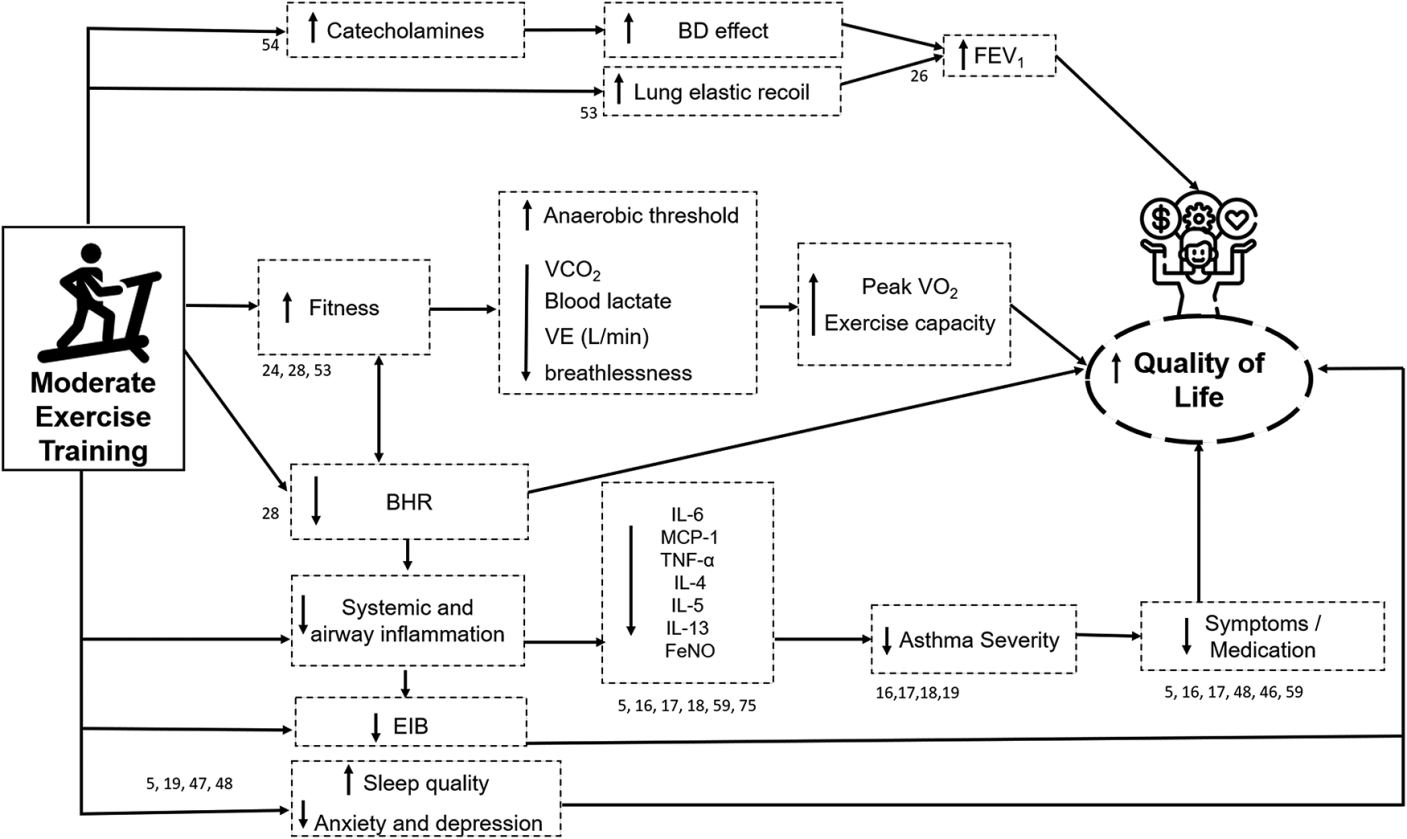
Illustrative figure of the effects of moderate exercise training in individuals with asthma. The numbers near the arrows and boxes represent the reference numbers that has demonstrated each effect. BD: Bronchodilator; FEV_1_: Forced expiratory volume in the first second; VCO_2_: Carbon dioxide production; VE (L/min): Minute Ventilation (Liters per minute); Peak VO_2_: Peak oxygen consumption; BHR: Bronchial hyperresponsiveness; EIB: exercise-induced bronchoconstriction; IL: interleukins; MCP-1: monocyte chemoattractant protein-1; TNF-α: Tumor Necrosis Factor, FeNO: Fractional Exhaled Nitric Oxide; Icons made by Freepik from www.flaticon.com.

Clark and Cochrane (1990) (25) demonstrated that moderate physical exercise for three months improves fitness and cardiorespiratory performance in patients with asthma. The improvement in the physical conditioning was observed by a significant increase in the maximal oxygen consumption, oxygen pulse, and anaerobic threshold and a reduction in breathlessness, blood lactate levels, carbon dioxide production, and minute ventilation during submaximal exercise. After that, systematic reviews have demonstrated that physical training effectively increases maximal oxygen consumption (23, 26) after physical training. Valkenborghs et al. (2022) (27) showed in a systematic review that aerobic exercise, in particular, improves cardiorespiratory fitness (peakVO_2 _= 3.1 ml/kg/min [1.9–4.3]; mean [95% CI]) and functional fitness (walking distance: MD 41 m [27–54]). It is suggested that exercise training may also reduce the perception of breathlessness through several mechanisms, including the respiratory muscles strengthening, leading to a greater maximum exercise effort (23, 24). Furthermore, multiple linear regression models revealed that changes in airway hyperreactivity contributed to improved exercise capacity (28) (Figure 2).

### Quality of life (QoL)

QoL can be significantly impaired in patients with asthma. Impaired QoL has been associated with several factors, including advanced age, increased asthma severity, poor asthma control, low education level, and low socioeconomic status (29). Asthma severity has been associated with worse QoL due to excessive symptoms, frequent and life-threatening attacks, increased comorbidity burden, and high pharmacological treatment dependence (30) (Figure 1). On the other hand, exercise training has been associated with improved QoL and has been proposed as adjunctive therapy for asthma in clinically stable patients (5, 24, 26). A recent systematic review included 22 studies, and the authors established that exercise training improves QoL and other health outcomes in patients with asthma (26). However, the meta-analysis pooled HRQOL and ACQ as measures of QoL, and most studies used ACQ rather than a QoL tool. Consequently, although the analysis was statistically significant, it limits the ability to demonstrate that exercise improves QoL. In addition, this review did not analyze the data using the Asthma Quality of Life Questionnaire (AQLQ) of two studies (31, 32) that were excluded without explanation. On the other hand, two other recent systematic reviews with meta-analysis showed a significant difference in favor of exercise in patients with asthma (33, 34). Feng et al. (2021) (33) included four studies (198 participants) and demonstrated an improvement in the total AQLQ score in favor of the patients with asthma that performed exercise training without statistical heterogeneity in the analysis. Furthermore, Zhu et al. (2022) (34) included nine studies in their meta-analysis and also demonstrated an improvement and reached the minimal clinically significant difference in QoL after aerobic exercise (AQLQ or pediatric AQLQ evaluated QoL).

The mechanisms involved in improving QoL may be associated with improvements in all domains of the AQLQ, including physical limitations, symptom frequency, socioeconomic conditions, and psychosocial health. Thus, QoL in patients with asthma can be assessed using either biological or clinical indicators (26). Multiple linear regression models have shown that improved BHR and lung function contribute significantly to improved QoL after exercise training (28). In addition, changes in FEV_1_ may partially explain the improvement in QoL in these individuals (28) after exercise training. In addition, symptom reduction and medication use can influence the improvement in QoL (24, 28). Furthermore, it is known that exercise training is associated with a decrease in systemic inflammation (17), which may contribute to reducing asthma severity (28), leading to a beneficial effect on QoL; as previously mentioned, increased asthma severity is associated with worse QoL. Finally, Chandratilleke et al. (2012) (24) suggest that, although the beneficial effects of exercise training on QoL could be a translation of improved cardiopulmonary fitness, further studies are required to evaluate this aspect in detail (Figure 2).

### Anxiety and depression

According to the World Health Organization (WHO), around 10% of the world's population is affected by depression and/or anxiety (35). Anxiety and depression have been linked to asthma (36–39) and are particularly associated with difficult-to-control asthma (40). A previous study showed that the prevalence of anxiety (30%) was greater in people with asthma, while depression (9%) was similar compared with people without asthma (41). Asthma symptoms result in sleep disturbances, irritability, and anxiety (42), affecting patients' QoL. In this perspective, Lavoie et al. (43) (2006) evaluated 504 adults with asthma and reported that depression and anxiety were associated with worse QoL and poorer asthma control (Figure 1). However, in patients without symptoms, QoL was similar to or even better than the population average (44).

Aerobic training is a non-pharmacological strategy that promotes a more significant reduction in the symptoms of anxiety and depression in patients with asthma (5). Mendes et al. (20) (2010) verified that in patients with moderate or severe asthma, an aerobic training program improved QoL and reduced symptoms of anxiety and depression. In addition, the highest anxiety and depression symptoms are associated with worse QoL (43). It is suggested that physical training reduces asthma symptoms, improving the QoL.

### Sleep quality

Sleep disturbance is common in patients with asthma and is associated with worse asthma control and QoL (45) (Figure 1). For example, Alanazi et al. (46) (2021) included a large number of patients with asthma, 66% had poor sleep quality, 43% were at high risk for obstructive sleep apnea, 25% had excessive daytime sleepiness, and 46.5% had clinically significant insomnia. Furthermore, this study also demonstrated that poor sleep quality was less common in patients with well-controlled asthma than those with partially controlled or uncontrolled asthma (37%, 78%, and 82%, respectively) (46).

In contrast, physical training is associated with reduced nocturnal symptoms and improved sleep quality in patients with asthma (47, 48). For instance, a systematic review by Francisco et al. (47) (2018) reported that supervised physical exercise for at least 12 weeks reduced nocturnal asthma prevalence, improved subjective sleep quality (Figure 2), and reduced nocturnal symptom frequency in non-obese adults with asthma. In addition, the authors reported that ten weeks of physical training reduced the prevalence of nocturnal asthma and was associated with a clinically significant reduction in nocturnal symptoms and enhanced subjective sleep quality in obese adults with asthma (47).

### Lung function

In early studies assessing the benefits of exercise training in patients with asthma, no effect was demonstrated on lung function (10); however, these studies presented small sample sizes. Systematic reviews have suggested an improvement in the lung function post-intervention (4, 5, 26, 28), including FEV_1_, forced expiratory flow at 25%–75% (FEF_25%−7%_), forced vital capacity (FVC), and peak expiratory flow rates (PEFR) (49). A recent systematic review by Hansen et al. (4) (2020) showed that FEV_1_ slightly improved in patients who underwent exercise training.

In contrast, other studies reported little or no effect on pulmonary function after physical training in patients with asthma subjected to exercise training (24). Careful evaluation of the studies included in the Hansen et al. meta-analysis showed that two out of ten included studies (31, 50) reported improved lung function in all individuals who underwent exercise training. In addition, in one of these studies (31), the individuals with asthma were obese (body mass index (BMI) ≥ 35 kg/m^2^), and they lost weight after the intervention; however, lung function was expressed as a percentage favoring the improvement in results. Consequently, we can suggest that the improvement in lung function observed in this systematic review may be distorted by including a very heterogeneous population and, more specifically, by weight loss rather than exercise training (51).

Shi et al. (26) also evaluated the effect of exercise training on lung function in a systematic review with a Bayesian meta-analysis. The authors expressed FEV_1_ and FVC in liters and the percentage of the predicted and the FEV_1_/FVC ratio. The only improvement observed was the FEV_1_ in the percentage of the predicted in favor of the physical exercise group. Interestingly, in this meta-analysis, in seven out of eight studies (87%), patients performed exercise training, and in one study, the intervention was an increase in physical activity. Taken together, these results suggest that the mechanisms underlying lung-function improvement depend on the asthma phenotype. The improvement in lung function in obese individuals with asthma appears to be related to a reduction in body weight. In contrast, in non-obese individuals with asthma, the improvement seems to be related to an improvement in FEV_1_, which might be explained by the bronchodilator effect of aerobic exercise (52). In addition, physical training can improve pulmonary elastic recoil, increasing pulmonary capacity (53) (Figure 2).

### Bronchial hyperresponsiveness (BHR) and EIB

BHR is a heightened bronchoconstrictive response to airway stimuli and complements EIB (Figure 1). The overlap between these two mechanisms and their association with airway inflammation remains unclear. Although BHR and EIB are considered pathophysiological hallmarks of asthma, these properties of the airway are dynamic because their severity and presence can vary over time with disease activity, triggers, or specific exposure. A deficiency in the catecholamine release during exertion has been suggested to explain the EIB in asthma patients (54).

In the BHR tests, bronchoconstriction is triggered by inhaled substances, such as methacholine and histamine, which act on the smooth airway muscles. A meta-analysis performed to evaluate BHR in response to inhaled substances revealed a tendency to favor exercise training over controls (28). Furthermore, three studies comparing BHR after exercise training demonstrated a reduction in BHR in those who exercised compared to controls (Figure 2). This result is supported by the analysis of percentage changes in all five controlled-exercise training groups, showing a significant average improvement of 53%. These impressive results demonstrate that exercise training affects airway response in patients with asthma. As previously mentioned, this can occur either by improving the modulation of the sympathetic–parasympathetic nervous system (catecholamine deficiency hypothesis) or by reducing airway inflammation (Th2- or non-Th2-mediated). However, the effects of exercise training on the BHR in obese patients have never been assessed. Thus, its effect on non-Th2-mediated inflammation remains to be confirmed. Nevertheless, independent of the mechanism, improvement in BHR after exercise training can play an important role in reducing symptoms and improving clinical control.

In contrast, a meta-analysis assessing the effect of exercise training on EIB did not show a difference between those who exercised and controls. Considering that BHR and EIB are airway responses to external stimuli, these results seem contradictory; however, this difference can be explained by several factors. First, there is a difference in the standardization of both tests (BHR and EIB). The BHR test is much better standardized; consequently, investigators can proceed with similar protocols, and fewer variables have been observed (55). Although recommendations for standardization of EIB challenges exist (56), studies have included different tests to assess it (57, 58). The only meta-analysis evaluating the role of exercise training on EIB used a coefficient of variation of 21%, a variability value that might mask real changes (28). Second, the discrepancy in improvement in BHR but not in EIB after exercise training may also result from the fact that both responses are triggered by different mechanisms. BHR tests assess the effect on airway smooth muscle contraction, whereas the EIB test induces bronchoconstriction indirectly by triggering the release of mediators. Consequently, the EIB challenge depends more on environmental factors, such as air temperature, humidity, pollution, and exercise intensity.

### Medication

It is expected that a change in inflammation accompanies changes in BHR and EIB. This hypothesis is supported by studies that observed an improvement in inflammatory variables after physical training (18, 59). From a clinical point of view, a reduced airway response strongly suggests that exercise training may have an anti-inflammatory effect in patients with asthma. These results are supported by evidence that exercise training can reduce the consumption of bronchodilators (58) and inhaled and oral corticosteroids (60). In a previous study, 52% of patients reported a reduction in inhaled corticosteroid use after exercise training (57), and three studies reported a decrease in systemic corticosteroid consumption (61–63). Unfortunately, none of these studies assessed parameters related to inflammation. In addition, the effect of exercise on medication consumption has been less assessed in the last years because several studies have tried to evaluate the effect in systemic and airway inflammation, and such assessment can only be studied without changing pharmacological treatment (17, 31, 48, 59).

### Inflammation

Asthma pathogenesis has not been fully understood (64, 65), and efforts have focused on identifying biomarkers applicable to clinical practice, and several allergic inflammation markers have been proposed (e.g., IgE, eosinophilia, fractional exhaled nitric oxide [FeNO]). In addition, T helper-2 (Th2) cell-mediated biomarkers of allergic response have also been characterized. Th2-mediated inflammatory cytokines include interleukins (ILs) such as IL-4, IL-5, IL-9, and IL-13 (Figure 1). In a recent study, Freeman et al. (2020) (16) evaluated the anti-inflammatory effects of exercise training in patients with asthma. They observed a reduction in the blood levels of IL-4, IL-5, IL-13, and m*onocyte chemoattractant protein*-*1 (*MCP-1) (Figure 2) and an improvement in bronchial hyperreactivity (BHR) and FEV_1_. Previous studies have shown that regular physical exercise reduces pulmonary inflammation in patients with asthma, assessed by a reduction in the eosinophil count (59, 66) and FeNO levels (18, 59). Hansen et al. (2020) (4) performed a meta-analysis and did not observe a significant change in airway inflammation. However, a small number of studies were included, and the FeNO levels, an indirect airway inflammation marker, were assessed in five out of six studies. In addition, both asthma severity and the biomarker were heterogeneous, which may have contributed to the lack of difference between groups.

Obesity has been associated with asthma with normal or low FeNO levels. However, among patients with asthma and high FeNO levels, obesity is associated with increased asthma severity. Obese individuals may have elevated levels of pro-inflammatory cytokines, which are associated with the inflammatory response of the airways, resulting in the worsening of asthma symptoms. The non-Th2-mediated phenotype is mainly related to neutrophilic inflammation and may be associated with the deregulation of the innate immune response, including abnormalities in the regulation of the phyla response and the type of conception *via* IL-17-dependent pathways (67, 68). The cytokines that may be associated with the non-Th2-mediated systemic inflammatory process are IL-2, IFN-*γ*, IL-12, and TNF-*β* (69) (Figure 1).

Elevated leptin concentrations are associated with deficient lung function in patients with asthma (70). In obese patients, pro-inflammatory cytokines, such as IL-1β, IL-6, and TNF*α*, favor the pathogenesis of asthma (71). In addition to contributing to metabolism, leptin influences the control of the vascular, hematopoietic, reproductive, and immune systems. Thus, physical exercise can increase or reduce immune responses in patients with asthma according to the intensity, duration, and frequency of training (72, 73). Scott et al. (2013) (74) showed a significant reduction in the sputum eosinophils after exercise training, indicating that exercise may be associated with an anti-inflammatory effect (Figure 2). Furthermore, Moraes-Ferreira et al. (2022) (75) found a reduction in IL-4, IL-5, IL-6, IL-13 and TNF-α cytokines in the plasma of patients with asthma undergoing aerobic training.

## Current physical training recommendations for patients with asthma

Physical training is considered an important component of the pulmonary rehabilitation program in patients with asthma, recommended by the leading international consensuses (76). It is important to emphasize that the best physical training model is one in which the patient best adapt and feels conformable performing it. In addition, we describe how an aerobic training session should be proposed for patients with asthma. These recommendations were described based on previous studies that carried out aerobic training in patients with asthma, demonstrating positive effects (6, 31, 77–79). (Table 1). People with asthma can safely perform resistance training to improve muscle fitness which also has a lower risk of developing EIB (80). Olenich et al. (2017) (81) verified that resistance training improves the clinical control of patients with asthma. Furthermore, the association of resistance training with aerobic exercise improves symptoms of depression and sleep efficiency in obese people with asthma (48). The recommendations for resistance training are described in Table 1 (48, 80).

**Table 1:** Proposed Aerobic Training Protocol for patients with asthma.

Phases	Composition for physical training		Duration (minutes)
**Initial assessment**	Pulmonary auscultation.Evaluate PEF, BP, perceived exertion (Borg scale), and HR.		5 to 10
	Assess PEF, BP, perceived exertion (Borg Scale), and HR.		
	SpO_2_ verification.		
**Warm-up**	Types of warm-up exercise: walking, cycling, or recreational games.		
	Intensity: 50% of peakVO_2_or HRmax.		
	Equipment or resources: cycle ergometer, elliptical machine, treadmill, free exercising.		
	Importance of warm-up: avoid triggering EIB.		
**Physical conditioning**	Types of aerobic exercise: walking, running, cycling or swimming.		
	Constant-Load Exercise Intensity: 60 to 80% of peakVO_2_ or HRmax. 20 to 30 minutes	Interval Aerobic Exercise Intensity: 80% to 140% Wmax based on CPET. HIIT: 30 seconds/Recovery 30 seconds (active exercise at 40% Wmax)	20 to 30
	Frequency: 2 to 3 times a week.		
	Monitoring: HR, SpO_2_, perceived exertion.		
	Equipment and resources: bicycle, treadmill, swimming pool, outside free area for exercising, frequency meter, oximeter, subjective perception scale to assess dyspnea and fatigue (Borg scale).		
	Optional exercise: Associate resistance training Muscle groups: pectoral, deltoid, quadriceps, and hamstrings. Reps:10–15 (2 to 4 sets). Interval: 3–4 minutes rest between sets Borg: 5–6 (10-point rating of perceived exertion scale)		
**Cool-down**	Types of exercises to cool-down: walking, cycling.		5 to 10
	Intensity: 50% of peakVO_2_or HRmax (Light).		
**Final evaluation**	Evaluate PEF, BP, Borg Scale, HR and Pulmonary auscultation.		5 to 10
	Warning: care must be taken to know that the PEF does not show a drop ≥ 10% of the value obtained in the initial assessment.		

PEF = Peak expiratory flow; BP = Blood pressure; HR = Heart rate; BD = short-acting bronchodilator (400 μg) (GINA, 2022); peakVO_2_ = peak oxygen consumption; HRmax = Maximum heart rate predicted for age (ACSM, 2021); SpO_2_ = Peripheral oxygen saturation; EIB = exercise-induced bronchoconstriction.

The high-intensity interval training (HIIT) has also been shown to induce beneficial effects on asthma control, exertional dyspnea, and anxiety in individuals with moderate to severe asthma (82, 83). Aparecido da Silva et al. (84) compared constant-load exercise vs. HIIT to improve dyspnea symptoms and clinical control in adults with moderate-to-severe asthma. Exercise training was performed twice a week for 12 weeks for 24 sessions. HIIT sessions lasted 40 min (5 min of warm-up, 30 min of exercise, and 5 min of cool-down) and were performed on a cycle ergometer. HIIT was performed in bouts, with the workload (maximal exercise intensity [Wmax]) based on the cardiopulmonary exercise testing (CPET). The result of this study suggests that constant-load exercise and HIIT effectively improve aerobic fitness in asthma. Furthermore, HIIT was more effective than constant-load exercise in reducing dyspnea levels, lower limb fatigue, and increasing physical activity levels.

## Breathing exercises

Breathing exercises should be offered to all patients with asthma with symptoms or impaired quality of life despite standard care. In addition, breathing exercises can improve patient-reported outcomes and psychological status. The most frequently investigated training techniques were breathing exercises administered by physical therapists and alternative breathing techniques such as the Buteyko method and yogic breathing (3). All breathing exercises have some features in common: stimulating stimulus of nasal and diaphragmatic breathing, increasing expiratory time, slowing breathing flow, and regulating breathing rhythm.

Evaristo et al. (6) compared the effects of aerobic training vs. breathing exercises in clinical control, quality of life, exercise capacity, and airway inflammation in outpatients with moderate to severe asthma. Breathing exercise program was based on Pranayama Yoga breathing technique. Aerobic training or breathing exercise programs presented similar results in asthma control, quality of life, asthma symptoms, psychological distress, physical activity, and airway inflammation.

## Research priorities for the area's future

Physical training in adults with asthma has been a highly discussed topic worldwide in recent years. This review highlights the benefits of exercise and provides essential information to facilitate clinicians' approach to improving physical training in this population. Based on the presented evidence, exercise should undeniably become important in asthma treatment. However, new horizons are emerging and should be explored, such as behavioral intervention to increase physical activity (85, 86) and understanding the better intervention for each asthma phenotype. In addition, it would be important to understand the effects of these interventions in the medium and long term and the effects of online interventions in this population. Finally, studies comparing the effects of exercise training in adults and children with asthma are needed as well as, discussing add-on strategies to physical training in this population.

## Conclusions

Asthma is associated with increased anxiety and depression symptoms, medication use, and reduced lung function, sleep quality, and physical activity levels, leading to impaired quality of life. In contrast, exercise training improves almost all outcomes in patients with asthma. This review presents the effects of exercise training on clinical and pathophysiological asthma outcomes. Moderate exercise training has been associated with a reduction in pulmonary and systemic inflammation, bronchial hyperresponsiveness, and exercise-induced bronchoconstriction. In addition, we report the benefits in asthma symptoms, medication use, anxiety and depression symptoms, lung function, exercise capacity, and sleep quality. This mechanism of improvement triggered by exercise training has an effect on the clinical condition of patients with asthma, leading to an improvement in quality of life. The most commonly used exercise strategy is moderate aerobic and breathing exercise; however, other strategies, such as high-intensity interval training, have shown promising effects for patients with asthma.
